# White matter endophenotypes and correlates for the clinical diagnosis of autism spectrum disorder

**DOI:** 10.1093/scan/nsy048

**Published:** 2018-06-22

**Authors:** Bun Yamagata, Takashi Itahashi, Motoaki Nakamura, Masaru Mimura, Ryu-Ichiro Hashimoto, Nobumasa Kato, Yuta Aoki

**Affiliations:** 1 Department of Neuropsychiatry, Keio University School of Medicine, Tokyo, Japan; 2 Medical Institute of Developmental Disabilities Research at Showa University, Tokyo, Japan

**Keywords:** ASD, bootstrapping, diffusion tensor imaging, endophenotypes, siblings

## Abstract

Since prior diffusion tensor imaging (DTI) studies reported no significant differences in white matter organizations between individuals with autism spectrum disorder (ASD) and their unaffected siblings, the neural correlates for developing a clinical diagnosis among people with endophenotypes remain undetermined. We obtained DTI data from a total of 60 participants consisting of 30 people with endophenotypes and 30 people without. We first followed a conventional approach by comparing individuals with ASD and their unaffected siblings. Using region-of-interest approach, we then performed bootstrapping to examine whether the differences in white matter organizations between individuals with ASD and their unaffected siblings were substantially large, considering the distribution of differences between typically developing (TD) siblings. Conventional approaches revealed no significant differences in white matter organizations between individuals with ASD and their unaffected siblings. Bootstrapping revealed a significantly large difference in axial diffusivity in the left stria terminalis between individuals with ASD and their unaffected siblings after accounting for the distribution of differences in axial diffusivity among TD siblings (99.998 percentile). The results remained significant after controlling for multiple comparisons with Bonferroni method. We assumed that one aspect of this tract was associated with the development of a clinical diagnosis.

## Introduction

### Autism spectrum disorder and white matter

Autism spectrum disorder (ASD) is a developmental disorder characterized by social interaction deficits and restricted and repetitive behaviors (American Psychiatric Association, 2013). Neuroimaging studies have shown a miswiring connectome among individuals with ASD (Di Martino *et al.*, 2014b; Ecker and Murphy, 2014; Ameis and Catani, 2015). Diffusion tensor imaging (DTI) is a modality for examining connectivity in the brain, and DTI studies have identified atypical structural connectivity in individuals with ASD (reviewed in Travers *et al.*, 2012; Aoki *et al.*, 2013). Although some results varied, the studies consistently showed atypical DTI parameters, including fractional anisotropy (FA), radial diffusivity (RD), mean diffusivity (MD) and axial diffusivity (AD), among individuals with ASD (Itahashi *et al.*, 2015; Catani *et al.*, 2016; Aoki *et al.*, 2017).

### Endophenotype

Endophenotypes are heritable and measurable components that are between diseases and genes (McGuffin *et al.*, 1987). Given that ASD shows heritability (Hallmayer *et al.*, 2011), endophenotypes emerge in the siblings of individuals with ASD (Lisiecka *et al.*, 2015; Chien *et al.*, 2017), regardless of their diagnostic status. Structural connectivity in the brain is one example of an ASD endophenotype (Gottesman and Gould, 2003). Indeed, prior DTI studies have revealed that individuals with ASD share alterations with their unaffected siblings (Barnea-Goraly *et al.*, 2010; Jou *et al.*, 2016; Chien *et al.*, 2017), supporting the existence of a white matter ASD endophenotype. Intriguingly, no differences in any DTI parameters were observed between individuals with ASD and their unaffected siblings (Barnea-Goraly *et al.*, 2010; Jou *et al.*, 2016; Chien *et al.*, 2017), leaving the neural correlates for the development of ASD among individuals with endophenotypes undetermined. However, identifying the neural basis for the development of a clinical diagnosis of ASD according to endophenotypes is informative because it may provide a clue to understanding the mechanism of the development of ASD among people with endophenotypes.

### Typical variance of brain volume

The brain shows substantial growth after birth. Although the brain is under a strong genetic influence, interactions among genes and environmental factors impact growth (Peper *et al.*, 2007). The influence of these interactions is particularly evident in white matter (Peper *et al.*, 2007) and varies regionally within the brain (Kochunov *et al.*, 2015). Thus, the variance of DTI parameters differs both within the brain and across individuals.

### Aim of this study

The aim of this study is to examine the neural correlates for an ASD clinical diagnosis among people with ASD endophenotype. To achieve this goal, we enrolled not only individuals with ASD and their unaffected siblings but pairs of typically developing (TD) siblings. The enrollment of pairs of TD siblings is motivated by the following two concerns. First, difference between individuals with ASD and their unaffected siblings may be influenced by both similarity between siblings and ASD endophenotype. Second, we assume that the DTI parameters of individuals with ASD and their unaffected siblings may not be normally distributed because of ASD endophenotype and influence of clinical diagnosis. In other words, differences between individuals with ASD and their unaffected siblings may encompass neural correlates for the development of a clinical diagnosis. However, ASD endophenotype may impact the differences in DTI parameters between individuals with ASD and their unaffected siblings, compared with that of TD siblings without ASD endophenotype. Prior neuroimaging studies involving individuals with ASD and their unaffected siblings included only one group of TD individuals as a control. Thus, they did not account for the distribution of white matter differences between TD siblings. This might have resulted in an underestimation of the differences in white matter between siblings with and those without a clinical diagnosis of ASD. To overcome this potential drawback, the overarching goal of this study was to dissociate white matter correlates of diagnosis from ASD endophenotypes. To achieve this goal, this study examined the differences in DTI parameters between siblings discordant for ASD diagnosis while accounting for the variance of differences in parameters by enrolling pairs of TD siblings. To compare the results from our novel approach with those from prior studies, we first followed the conventional approaches to explore the differences in white matter between individuals with ASD and their unaffected siblings. Practically, we performed Tract-Based Spatial Statistics (TBSS) analyses contrasting individuals with ASD and their unaffected siblings. Second, using region-of-interest (ROI) analyses, we used paired *t*-tests to compare the DTI values between individuals with ASD and their unaffected siblings. Then, the similarity of white matter organizations between individuals with ASD and their unaffected siblings and the similarity between TD siblings were separately assessed using the intraclass correlation. Finally, as a novel approach, we implemented bootstrapping to examine the difference in mean DTI values between individuals with ASD and their unaffected siblings were substantially large when overlaid on the distribution of DTI parameter differences between TD siblings. To increase the homogeneity among participants, we recruited only adult males without intellectual disabilities. Furthermore, given that motion influences the DTI results, we ensured that the participants were only permitted a small amount of head motion and that head motion was accounted for in all the analyses.

## Materials and methods

### Participants

We analyzed data from 60 male individuals consisting of 30 pairs of biological siblings. In total 15 pairs of participants were discordant for ASD. The other set of data consisted of 15 pairs of TD siblings. The diagnosis of ASD was based on the DSM-IV-TR (American Psychiatric Association, 2000). Furthermore, the parents of the participants with ASD and their siblings were interviewed using the Autism Diagnostic Interview-Revised (ADI-R) (Tsuchiya *et al.*, 2013) to confirm the absence of a diagnosis of ASD in the unaffected sibling. Handedness was evaluated using the Edinburgh Handedness Inventory (Oldfield, 1971). The intelligence quotient (IQ) was assessed using either the Wechsler Adult Intelligence Scale-Third Edition or the WAIS-Revised (Wechsler, 1981, 1997). All the participants completed the Japanese version of the Autism-Spectrum Quotient (Wakabayashi *et al.*, 2006). Two of the participants had psychiatric comorbidities: one had attention-deficit/hyperactivity disorder, and the one had learning disabilities. Five of the participants were taking medication at the time of scanning: three were taking benzodiazepine, three were taking anti-depressants, and one was taking a stimulant. The absence of an Axis I diagnosis per the DSM-IV-TR was confirmed with the Mini-International Neuropsychiatric Interview (Hergueta *et al.*, 1998). Further, the absence of a history of psychotropic medication use was required for inclusion in the TD group. The exclusion criteria for all the participants were known genetic diseases or an estimated full IQ of below 80. After a complete explanation of the study to the participants, written informed consent was obtained from all the participants. The study protocol was approved by the Ethics Committee of Showa University and was prepared in accordance with the ethical standards of the Declaration of Helsinki.

### Comparison of demographic characteristics

Demographic characteristics, such as age, full IQ, verbal IQ, performance IQ, and the Edinburgh Handedness Inventory, were compared between individuals with ASD and their unaffected siblings using paired *t*-tests. Furthermore, paired *t*-tests were performed to compare the AQ scores between individuals with ASD and their unaffected siblings. The differences in abovementioned demographic characteristics and the AQ scores were also examined between TD siblings (younger brothers vs. older brothers). Finally, we used analysis of variance (ANOVA) to examine differences in the AQ scores between individuals with ASD, their unaffected siblings, and all the TD (younger and older siblings combined groups). Statistical threshold for significance was set at P < .05 for all the comparisons.

### Data acquisition

All MRI data were acquired using a 3.0-T MRI scanner (MAGNETON Verio; Siemens Medical Systems, Erlangen, Germany) with a 12-channel head coil. Diffusion-weighted images were collected using an echo-planar imaging spin echo sequence with 10 *b0*-images and 65 directions of diffusion gradients (in-plane resolution, 2.0 × 2.0 mm; 2-mm slice thickness with no gap; repetition time, 13.7 s; echo time, 79 ms; b-value, 1000 s/mm^2^; matrix size, 100 × 100; phase encoding, anterior-to-posterior; 74 transversal slices; number of excitations, 1). The *b0* images were acquired in an intermittent manner, and the directions of the gradients were optimized according to a previously described method (Caruyer *et al.*, 2013).

### Pre-processing and head motion

Pre-processing and the analyses were performed using FMRIB Software Library, Version 5 (FSL; http://www.fmrib.ox.ac.uk). Pre-processing included eddy current and motion corrections. Our head motion index was the mean absolute inter-volume displacement with respect to the first image of each run; the root-mean-square deviation was calculated using the FSL rmsdiff function. Since motion introduces artifacts in the DTI findings (Yendiki *et al.*, 2014), we set the inclusion criterion for motion as a mean head motion <2.5 mm.

### TBSS pre-processing

First, the data were skull-stripped using the FSL Brain Extraction Tool and registered to the Montreal Neurological Institute 152 standard space using non-linear registration. The FA images were estimated using the function ‘dtifit’ implemented in the FMRIB’s Diffusion Toolbox (FDT) software, which is a part of the FSL. Then, all the FA images were averaged to create the mean FA image and skeleton, which represents the centers of the major tracts common to the group (Smith *et al.*, 2006). The FA threshold was set at 0.3 (Barnea-Goraly *et al.*, 2010). Other DTI parameters, including MD, RD and AD, were projected onto the mean FA skeleton. We conducted TBSS pre-processing for two groups: the first group included only individuals with ASD and their unaffected siblings, while the second group included all the participants.

### Statistical analysis

#### TBSS analyses

##### ASD vs unaffected siblings

To examine the difference in DTI parameters between individuals with ASD and their unaffected siblings, we conducted paired *t*-tests using the function ‘randomise’ implemented in the FSL. Age and motion were included as nuisance covariates. Statistical significance was set at a family-wise error rate (α = 0.05)-corrected Threshold-Free Cluster Enhancement *P* < 0.05, with a minimum cluster size of 10 voxels (Bloemen *et al.*, 2010; Aoki *et al.*, 2018).

#### ROI analyses

##### ROI setting

We overlaid the International Consortium for Brain Mapping DTI-81 atlas on the TBSS skeleton and created 48 ROIs focusing on the centers of the major tracts (Mori *et al.*, 2008). The DTI values were controlled for age and motion in the following ROI analyses.

##### Paired t-tests

For each ROI, we conducted a paired *t*-test to further examine the difference in DTI metrics between adult males with ASD and their brothers. The statistical threshold for significance was set at a two-tailed *P* < 0.001 (=0.05/48), correcting for multiple comparisons.

#### Intraclass correlation

##### Similarity between TD siblings

We performed intraclass correlation analyses between adult male typical siblings. To divide TD sibling pairs into two groups, we focused on their birth order. This is because the advanced paternal age is associated with ASD risk. In addition, the birth order may also matter although the statistically significant difference in ASD risk was shown in comparison between the first and the third or later born offspring (Reichenberg *et al.*, 2006; Durkin *et al.*, 2008). To examine similarity between males with ASD and their unaffected siblings, we also performed intraclass correlation analyses for each DTI metric for each ROI. We utilized the single fixed rater intraclass correlation coefficient (ICC) to compute the genetic contribution on the DTI (Budisavljevic *et al.*, 2016). Statistical significance was set at *P* < 0.001 (=0.05/48).

#### Bootstrapping

To evaluate the difference in DTI metrics between adult males with ASD and their unaffected brothers while accounting for the typical distribution of differences in DTI metrics between siblings, we performed bootstrapping. First, we randomly assigned each of the siblings into two TD groups and calculated the mean difference between the two groups, which provided the distribution of the differences in DTI parameters for a given ROI between TD siblings. Because of the stringent threshold for statistical significance (see below), we repeated this procedure with 100 000 iterations. Then, we overlaid the mean difference in DTI parameters between adult males with ASD and their unaffected brothers on the distribution of DTI parameters between TD siblings. The statistical threshold for significance was set at either above the 99.998 or below the 0.002 percentile, which was equivalent to a two-tailed *P* < 0.001 (=0.05/48).

## Results

### Participants’ characteristics

#### ASD vs unaffected siblings

There were no significant differences in age, full IQ, verbal IQ, performance IQ or handedness between individuals with ASD and their unaffected siblings. Individuals with ASD had significantly higher total and AQ subscale scores, compared with their unaffected siblings (Table 1). Unaffected siblings scored below the cutoff threshold of the ADI-R (Table 1).

**Table 1.** Characteristics of the participants

	ASD (*n* = 15)		Unaffected ASD siblings (*n* = 15)		Statistics^a^			TD^b^ (*n* = 15)		TD^c^ (*n* = 15)		Statistics^d^		
					df	*t*-value	*P*-value					df	*t*-value	*P*-value
	Mean	SD	Mean	SD				Mean	SD	Mean	SD			
Age (years)	28.3	6.1	28.0	7.3	14	0.45	0.657	28.4	6.5	25.1	5.3	14	5.62	<0.001
Full IQ	111.8	16.0	107.4	13.2	14	0.77	0.452	115.9	15.7	114.9	12.1	14	0.24	0.815
Verbal IQ	106.3	31.2	107.1	16.8	14	0.08	0.940	117.1	15.9	116.7	12.5	14	0.18	0.859
Performance IQ	108.1	16.4	105.3	9.8	14	0.62	0.547	109.4	13.6	107.7	7.9	14	0.42	0.678
Handedness	61.8	68.4	99.3	2.9	14	2.11	0.054	89.2	26.4	80.7	51.4	14	0.55	0.594
ADI-R^e^														
Social interaction	20.7	6.3	0.8	1.4	13	11.56	<0.001							
Communication (verbal)	12.6	5.0	0.4	1.1	13	8.83	<0.001							
RRB	3.9	2.0	0.0	0.0	13	7.02	<0.001							
AQ														
Total	34.2	6.2	19.4	6.9	12	6.27	<0.001	17.6	5.5	14.9	6.0	14	1.12	0.280
AS	7.4	1.6	3.9	1.8	12	6.30	<0.001	4.0	2.1	3.7	1.4	14	0.54	0.596
ATD	5.4	1.9	3.2	1.9	12	2.67	0.020	3.4	2.1	3.9	1.6	14	0.88	0.396
COM	7.6	1.6	3.6	2.6	12	4.16	0.001	3.1	2.5	2.6	2.3	14	0.50	0.625
IMG	6.0	2.0	4.1	1.4	12	2.31	0.040	3.7	1.7	2.8	1.7	14	1.26	0.229
SS	7.9	2.0	4.5	2.7	12	4.26	0.001	3.4	2.7	2.0	2.1	14	1.38	0.191
SES^f^	5.5	1.1	5.5	1.1	14	0.20	0.843	5.9	1.2	5.7	1.1	13	0.46	0.655
Mean head motion	1.5	0.2	1.5	0.3	14	0.12	0.906	1.6	0.5	1.5	0.1	14	0.95	0.356
Max head motion	2.3	0.3	2.5	1.0	14	1.05	0.313	2.3	0.8	2.2	0.2	14	0.85	0.407

a Statistics show the results of comparisons between adult males with ASD and their unaffected brothers.

b Older siblings.

c Younger siblings.

d Statistics show the results of comparisons between typical older and younger siblings.

e The ADI-R score was missing for one person.

f A higher score indicates a lower socioeconomic status (Okada *et al.*, 2014).

Abbreviations: AQ, autism-spectrum quotient; AS, attention switching/tolerance of change; ASD, autism spectrum disorder; ATD, attention to detail; COM, communication skills; IMG, imagination; IQ, intelligence quotient; RRB, restricted repetitive behaviors; SD, standard deviation; SES, socioeconomic status; SS, social skills; TD, typical development.

#### TD vs their TD siblings

There were no significant differences in full IQ, verbal IQ, performance IQ, handedness or AQ scores between the older and younger siblings (Table 1).

#### ASD vs unaffected siblings vs all TD

An ANOVA showed significant effects of group on the total and AQ subscores. Post-hoc analyses revealed that these effects were driven by the group of individuals with ASD. In terms of social skills subscale, the unaffected siblings of the individuals with ASD had significantly higher scores than the all-TD group combining both younger and older siblings (Table 2).

**Table 2.** Comparison of individuals with ASD, their unaffected siblings and TD participants

	Statistics^a^			
	df	*f*-value	*P*-value	Post-hoc
Age (years)	2, 57	0.37	0.693	
Full IQ	2, 57	1.61	0.209	
Verbal IQ	2, 57	1.92	0.157	
Performance IQ	2, 57	0.38	0.688	
Handedness	2, 57	2.73	0.073	
AQ				
Total	2, 55	41.55	<0.001	ASD>sib=TD
AS	2, 55	22.30	<0.001	ASD>sib=TD
ATD	2, 55	5.42	0.007	ASD>sib=TD
COM	2, 55	20.93	<0.001	ASD>sib=TD
IMG	2, 55	12.17	<0.001	ASD>sib=TD
SS	2, 55	20.96	<0.001	ASD>sib>TD
SES^b^	2, 56	0.31	0.732	
Mean head motion	2, 57	0.08	0.920	
Max head motion	2, 57	0.93	0.399	

a Results of a comparison of three groups, individuals with ASD, their unaffected siblings, and all TD participants.

b A higher score indicates a lower socioeconomic status.

Abbreviations: AQ, autism-spectrum quotient; AS, attention switching/tolerance of change; ASD, autism spectrum disorder; ATD, attention to detail; COM, communication skills; IMG, imagination; IQ, intelligence quotient; SD, standard deviation; SES, socioeconomic status; SS, social skills; TD, typical development.

### TBSS analysis

No significant differences in any of the DTI parameters were seen between the individuals with ASD and their unaffected siblings.

### ROI analyses

#### Paired t-tests

##### ASD vs unaffected ASD siblings

Paired *t*-tests showed no significant differences in any of the DTI parameters between individuals with ASD and their unaffected siblings. These results did not change when age and motion were not regressed out.

### Intraclass correlation

#### TD and TD siblings

**Fig. 1.**
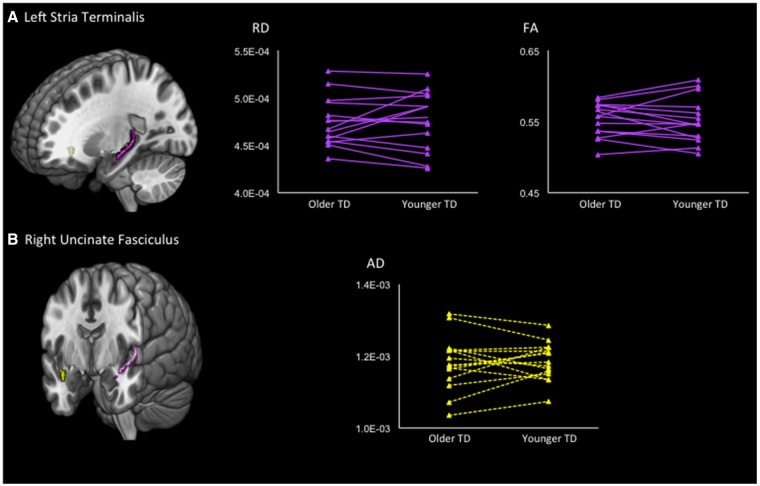
Results of intraclass correlation analyses between siblings with TD. The ROIs are shown in the left column. (A) Upper panel shows the left stria terminalis. In the right column, the line graphs show the RD and FA values of pairs of siblings with TD. The left dots of each line show the DTI values of the older siblings. Intraclass correlation analyses showed a statistically significant similarity in the mean RD (ICC = 0.772, *P* < 0.001) and FA (ICC = 0.704, *P* = 0.001). (B) Axial diffusivity in the right uncinate fasciculus exhibited a statistically significant intraclass correlation (ICC = 0.682, *P* = 0.002).

The intraclass correlation analyses showed a significant similarity in the left stria terminalis for RD (ICC = 0.772, *P* < 0.001) and a marginally significant correlation for FA (ICC = 0.704, *P* = 0.001) among typical siblings. The AD analysis showed a marginally significant intraclass correlation in the right uncinate fasciculus (ICC = 0.682, *P* = 0.002) (Figure 1).

#### ASD and unaffected siblings

**Fig. 2.**
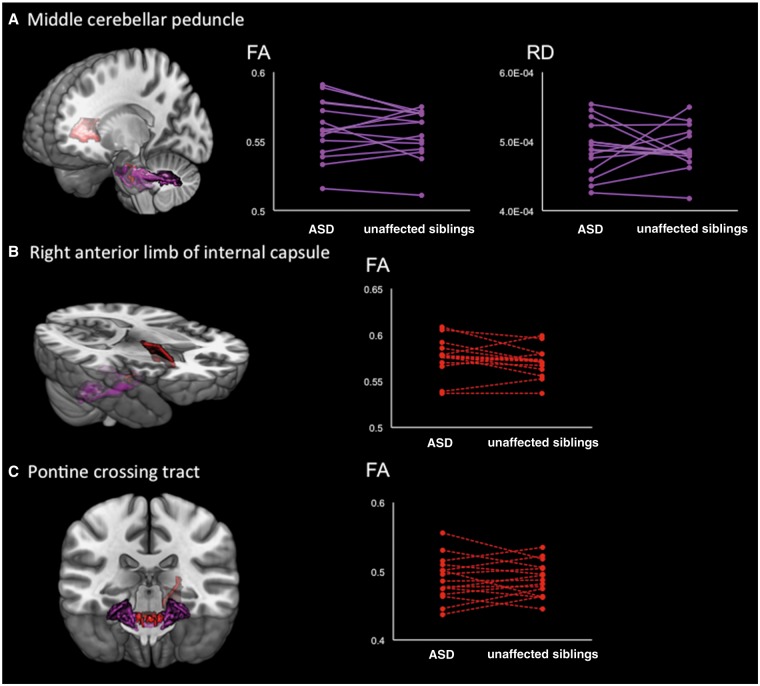
Results of intraclass correlation analyses between individuals with ASD and their unaffected brothers. (A) Upper panel demonstrates the results of intraclass correlation coefficients in the middle cerebellar peduncle. The mean FA (ICC = 0.756, *P* < 0.001) and RD (ICC = 0.748, *P* < 0.001) showed statistically significant similarities between individuals with ASD and their unaffected siblings. (B and C) Mean FA values in the right anterior limb of the internal capsule (ICC = 0.683, *P* = 0.002) and pontine crossing tract (ICC = 0.678, *P* = 0.002) show significant similarities at the trend level. The dots on the left tip of the lines represent individuals with ASD, while the dots on the right tip of the lines indicate their unaffected siblings.

Significant intraclass correlations between individuals with ASD and their unaffected siblings were observed for FA (ICC = 0.756, *P* < 0.001) and RD (ICC = 0.748, *P* < 0.001) in the middle cerebellar peduncle. Furthermore, the FA analyses showed a marginally significant intraclass correlation in the right anterior limb of the internal capsule (ICC = 0.683, *P* = 0.002) and the pontine crossing tract (ICC = 0.678, *P* = 0.002) (Figure 2).

### Bootstrapping

**Fig. 3.**
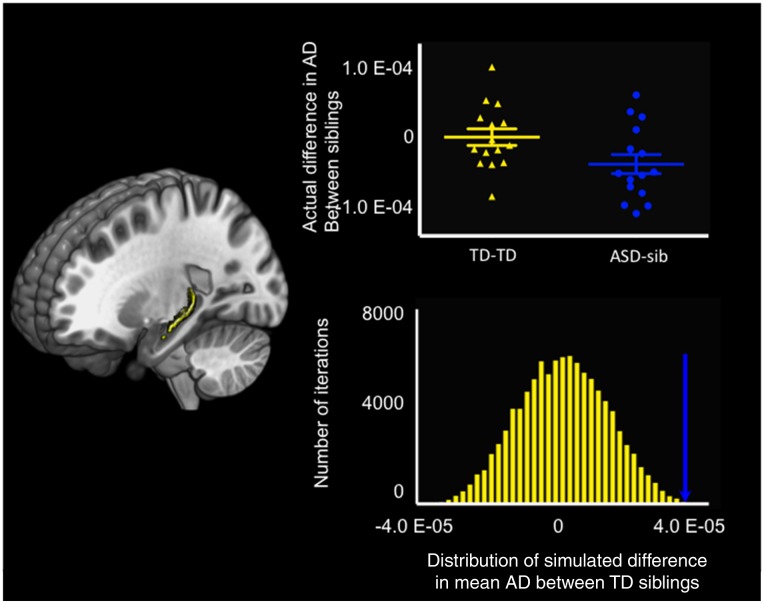
Results of bootstrapping. The left column shows the extension of the left stria terminalis. The upper subpanel in the left column demonstrates the actual difference in the mean AD values between TD siblings (shown in yellow) and the difference between individuals with ASD and their unaffected brothers (shown in blue). In the lower subpanel in the left column, the distribution of the differences in mean AD values between TD siblings as computed using bootstrapping with 100 000 iterations is shown in yellow. The blue arrow demonstrates the actual difference in mean AD values in the left stria terminalis between individuals with ASD and their unaffected brothers.

The AD analysis revealed a significantly large difference in the mean AD value between individuals with ASD and their unaffected siblings in the left stria terminalis (99.998 percentile). No other DTI metrics showed any significantly large differences in mean values between adult males with ASD and their unaffected brothers after accounting for the typical distribution of the mean differences in DTI parameters for each ROI between siblings (Figure 3). These results remained substantially unchanged when age and motion were not regressed out.

## Discussion

Utilizing DTI data from 30 pairs of siblings, we dissociated white matter neural correlates for the development of a clinical ASD diagnosis from the white matter ASD endophenotype, accounting for the distribution of differences in white matter organization between TD siblings. Our novel approach revealed that the left stria terminalis was related to the development of an ASD diagnosis. On the other hand, the results of conventional approaches in this study were consistent with the results of prior studies (Barnea-Goraly *et al.*, 2010; Jou *et al.*, 2016; Chien *et al.*, 2017). Specifically, comparisons between adult males with ASD and their unaffected brothers did not show any significant differences in white matter organization, regardless of whether a voxel- or cluster-level approach was used. Our novel framework suggested that such conventional approaches may underestimate differences in white matter organization between siblings discordant for ASD by not accounting for the typical distribution of responses to environmental factors of a given structure.

The stria terminalis is a bilateral structure connecting the bed nucleus of the stria terminalis (BNST) and the amygdala (Dumont, 2009). Although the function of the white matter part of the stria terminalis has not been fully elucidated, a variety of social behaviors, including social recognition and attachment, are related to both the BNST and amygdala (Insel and Young, 2001; Lebow and Chen, 2016). Research has also showed that these areas are largely influenced by genes and exhibit sexual dimorphism (Zhou *et al.*, 1995; Goldstein *et al.*, 2001). Despite the strong influences of genes, sex dimorphism in the BNST becomes substantial only during adulthood as a result of long-term interactions between genes and sex hormones (Chung *et al.*, 2002). Since the current study enrolled only adult males, the present finding that typically developing siblings exhibit a high degree of similarity in RD for this region may reflect a high contribution of genes and the similar accumulated response to sex hormones.

On the other hand, adult males with ASD and their unaffected brothers showed a substantially large difference in AD in the stria terminalis. Assuming that AD reflects axonal damage (Budde *et al.*, 2009), one potential interpretation of this result is that the difference in axonal damage may represent a consequence of the development of ASD. Another potential explanation is that the damage in this structure is related to a disorder-specific interaction between gene and sex hormones that may eventually lead to a clinical diagnosis of ASD. Intriguingly, longitudinal neuroimaging studies involving individuals with hormone replacement therapy have revealed that the white matter is vulnerable to the administration of sex hormones (reviewed in Brinton, 2016). We speculated that although the influence of sex hormones in this structure is consistent among people without ASD endophenotypes, reactions to hormones in this structure can vary among people with ASD endophenotypes. Sexual dimorphism of the BNST and amygdala, the vulnerability of white matter to sex hormones, and sex differences in ASD may support our speculation (Baron-Cohen *et al.*, 2005; Ruta *et al.*, 2011; Floris *et al.*, 2013; Nordahl *et al.*, 2015; Loomes *et al.*, 2017).

The middle cerebellar peduncle showed high similarity between individuals with ASD and their unaffected brothers but not between TD siblings. Since we enrolled pairs of TD siblings and did not have any reasonable means of selecting one sibling from each sibling pair, this study did not compare individuals with ASD and TD. However, the ASD-specific similarities of the DTI parameters in this tract may be consistent with the results of a previous report on DTI showing an abnormality in this tract among individuals with ASD (Catani *et al.*, 2008; Shukla *et al.*, 2010). Therefore, our findings for the middle cerebellar peduncle may represent white matter endophenotypes of ASD.

### Limitations

This study had several limitations. Because enrollment of TD siblings is the strength of the current study, we did not examine difference between individuals with ASD and TD like other studies with a larger number of participants. In addition, practical difficulties prevented us from enrolling monozygotic twins. Monozygotic twins have identical genes and are more suitable to the aim of this project. In addition, for the same reason, it was not possible to recruit siblings concordant for ASD, which may have enabled us to further confirm our findings of neural correlates for a clinical ASD diagnosis. Future studies involving multiple centers of investigation might be able to recruit a large enough sample of monozygotic twins who are either discordant or concordant for ASD. Second, although we adopted stringent thresholds for significance in novel and sound analyses, we cannot assume a causal relationship between the neuroimaging findings and the development of ASD. Furthermore, typical age for the onset of ASD is the first three years of life. Although ASD is believed to be a lifetime diagnosis, neuroimaging studies have shown atypical aging effect (Aoki *et al.*, 2012a; Kleinhans *et al.*, 2012; Di Martino *et al.*, 2014a), especially during childhood (Redcay and Courchesne, 2005; Aoki *et al.*, 2012b). Because we recruited only adults when the ASD brain does not change dramatically, the results of this study can be homogeneous. However, it cannot be generalized to children or adolescents, and the results from young children can directly address the development of the diagnosis (Hazlett *et al.*, 2017). Future research with a longitudinal design may overcome these limitations. Third, to increase the between-subject homogeneity, we included only males. This could explain why we successfully detected a difference in the stria terminalis, which is a sexually dimorphic structure. However, the results of this study cannot be generalized to females. Given the male-skewed prevalence of ASD, a larger number of collaborators is necessary to recruit female siblings discordant for ASD, compared with the recruitment of male siblings. Open data sharing is one potential solution (Milham, 2012).

## Conclusions

We utilized a novel framework to dissociate neural correlates for the development of a clinical diagnosis of ASD from neuroendophenotypes, recruiting not only siblings discordant for ASD but also pairs of typically developing siblings. The RD similarity in the left stria terminalis between TD siblings may represent genetic contribution; the AD in the structure may be related to the development of a clinical diagnosis of ASD among those who have ASD endophenotype.
